# Laparoscopic Distal Pancreatectomy with or without Preservation of the Spleen for Solid Pseudopapillary Neoplasm

**DOI:** 10.1155/2015/487639

**Published:** 2015-10-26

**Authors:** Tomohide Hori, Toshihiko Masui, Toshimi Kaido, Kohei Ogawa, Kentaro Yasuchika, Shintaro Yagi, Satoru Seo, Kyoichi Takaori, Masaki Mizumoto, Taku Iida, Yasuhiro Fujimoto, Shinji Uemoto

**Affiliations:** Department of Hepatobiliary Pancreatic Surgery, Kyoto University Hospital, Kyoto 606-8507, Japan

## Abstract

Solid pseudopapillary neoplasm (SPN) is a rare tumor of the pancreas. Laparoscopic distal pancreatectomy (DP) is a feasible and safe procedure, and successful spleen preservation rates are higher using a laparoscopic approach. We hypothesized that certain patients with SPN would be good candidates for laparoscopic surgery; however, few surgeons have reported laparoscopic DP for SPN. We discuss the preoperative assessment and surgical simulation for two SPN cases. A simulation was designed because we consider that a thorough preoperative understanding of the procedure based on three-dimensional image analysis is important for successful laparoscopic DP. We also discuss the details of the actual laparoscopic DP with or without splenic preservation that we performed for our two SPN cases. It is critical to use appropriate instruments at appropriate points in the procedure; surgical instruments are numerous and varied, and surgeons should maximize the use of each instrument. Finally, we discuss the key techniques and surgical pitfalls in laparoscopic DP with or without splenic preservation. We conclude that experience alone is inadequate for successful laparoscopic surgery.

## 1. Introduction

Epithelial neoplasms of uncertain differentiation, such as solid pseudopapillary neoplasm (SPN), pancreatoblastoma, and undifferentiated carcinoma, are rare tumors of the pancreas. Laparoscopic surgery for pancreatic tumors is well described [1–5] and certain patients with SPN are good candidates for laparoscopic surgery [6, 7]. However, few surgeons have reported laparoscopic distal pancreatectomy (DP) for SPN [6–8]. We discuss our surgical approach using laparoscopic DP with or without preservation of the spleen for two patients with SPN, with the key techniques and pitfalls. The surgical procedures were approved by our institutional ethics committee and review board, and both patients gave written consent for publication of the details of their cases.

## 2. Case Presentation

### 2.1. Case  1

**Figure 1:**
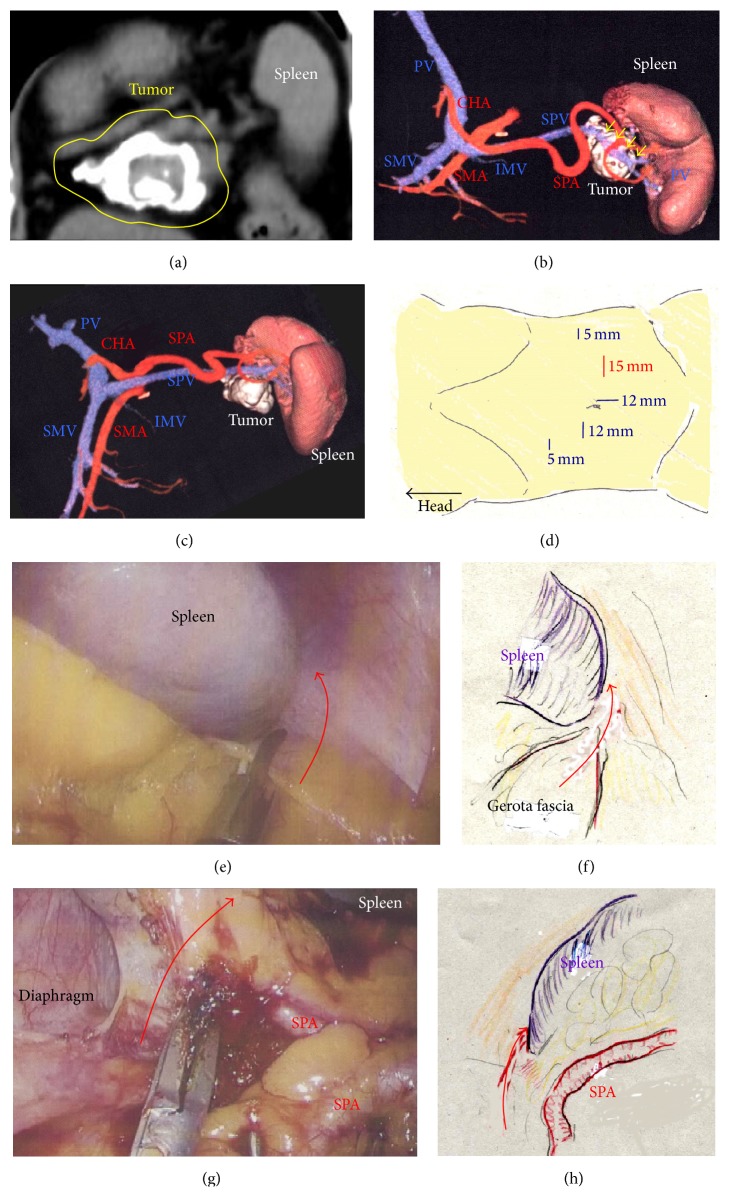
(a) The tumor surface was severely calcified (yellow circle). (b) Three-dimensional images revealed that the tumor involved the SPV (yellow arrows). (c) The SPA was not involved in the tumor. (d) A total of five ports were placed. (e) and (f) The inferior ligament of the spleen was cut (red arrow). (g) and (h) The superior ligament of the spleen was cut (red arrow). SPA, splenic artery; SPV, splenic vein.

**Figure 2:**
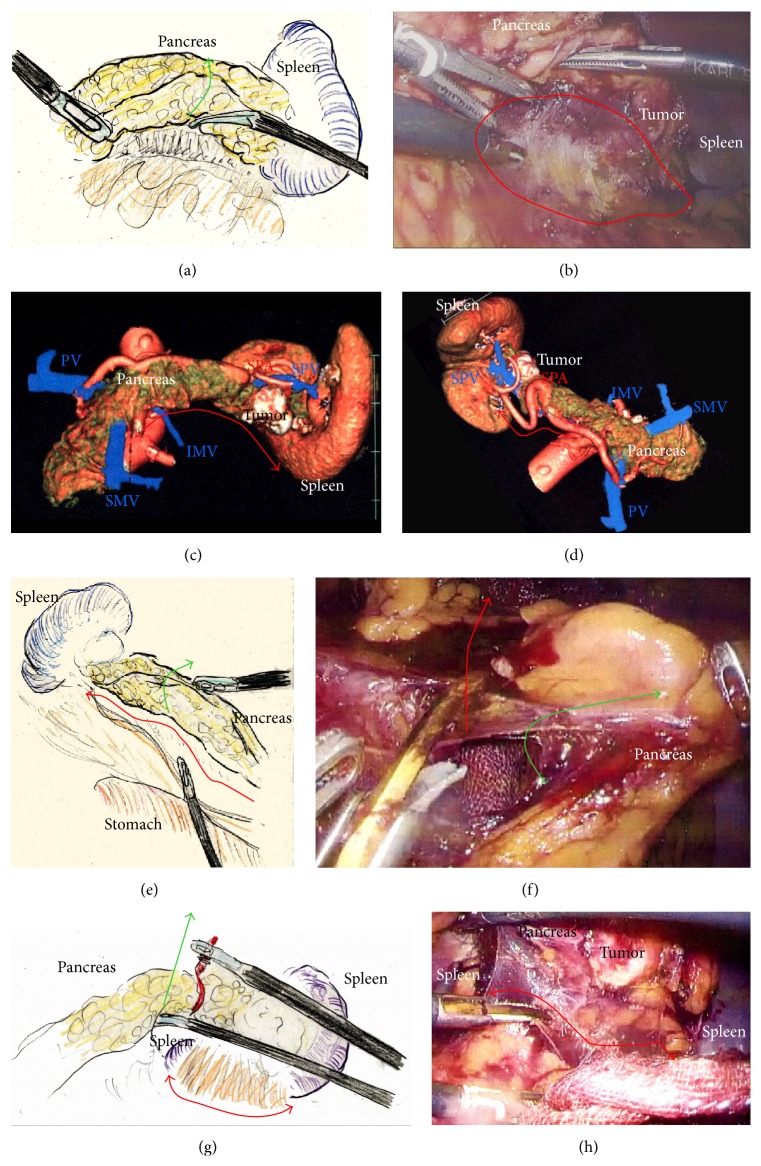
(a) The pancreas was retracted during a dorsal approach (green arrow). (b) During the dorsal approach, the distal pancreas was securely mobilized by dissecting the connective tissue (red circle). (c) Removal of the pancreas by a dorsal approach was simulated preoperatively (red arrow). (d) Removal of the pancreas by a ventral approach was also simulated preoperatively (red arrow). (e) and (f) The pancreas was retracted (green arrow) and then a ventral approach was recommended to complete the removal of the pancreas (red arrow). (g) and (h) The pancreas was retracted using the forceps shaft and/or tape (green arrow). Next, connective tissue and the thin membrane surrounding the spleen were cut using a dorsal approach (red arrow). The distal pancreas and spleen were then completely removed.

**Figure 3:**
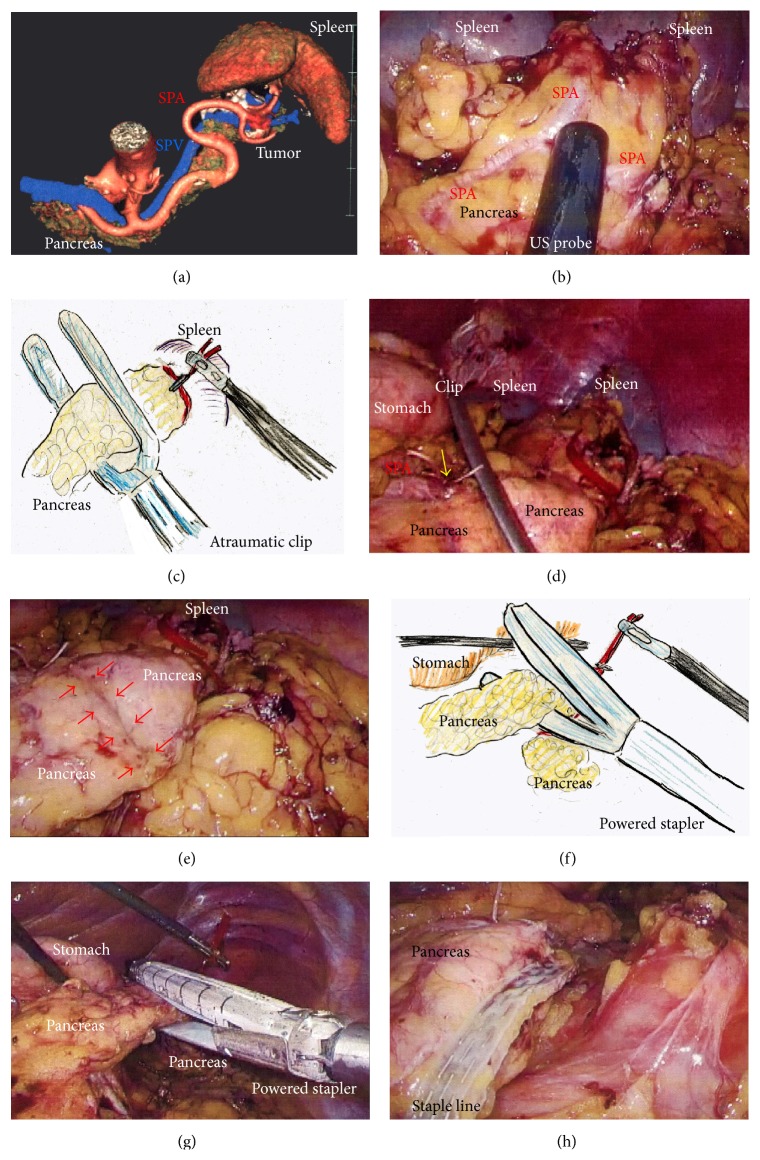
(a) Tumor location and anatomical landmarks were assessed preoperatively. (b) Ultrasound was performed to confirm the tumor location and to determine the cutting line. (c) Compression using an atraumatic clip was required before proceeding further because stapling was performed using a Covidien stapler. (d) The proximal SPA was ligated (yellow arrow) and the pancreas was compressed. (e) Compressed parenchyma was confirmed before proceeding further (red arrows). (f), (g), and (h) The pancreas and SPV were cut* en bloc* using the powered stapler; the pancreas was retraced with tape. SPA, splenic artery; SPV, splenic vein.

**Figure 4:**
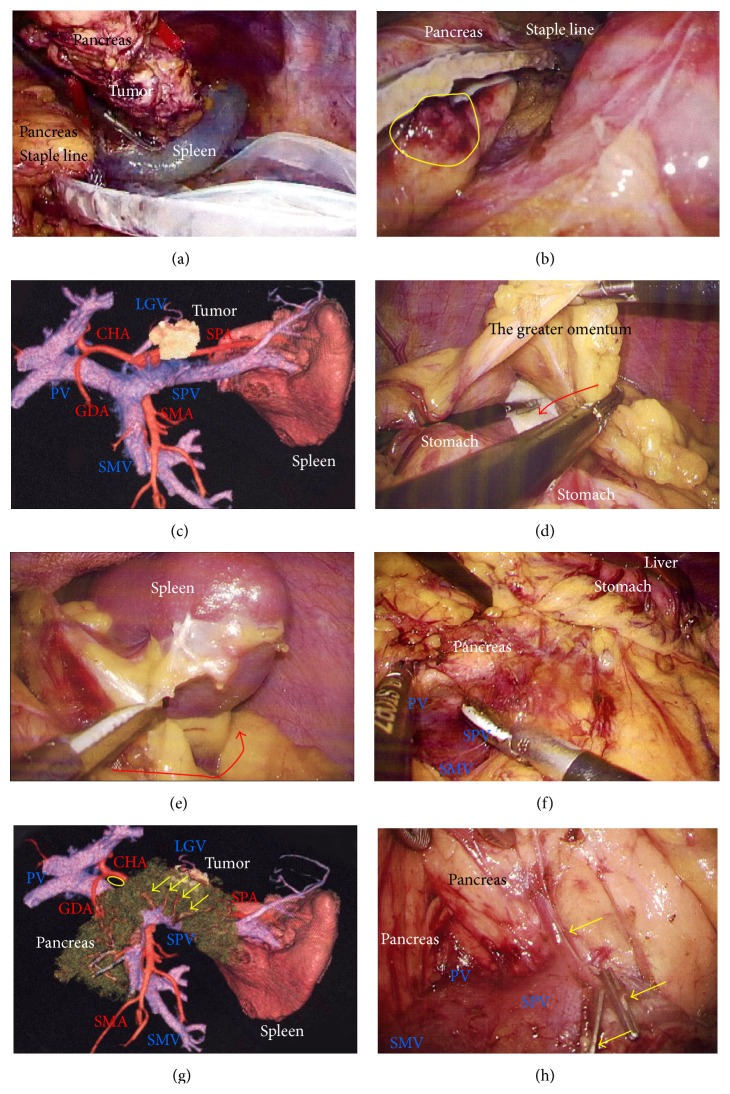
(a) The specimen was extracted through a 3 cm incision in an endobag to prevent dissemination by direct contact with the tumor. (b) The pancreatic stump and capsule were checked carefully. Subtle injury to the pancreatic membrane caused postoperative leakage of pancreatic fluid (yellow circle). (c) The tumor location and invasive signs were assessed carefully. (d) Gauze was placed on the splenic hilus as a landmark. The greater omentum was then cut (red arrow), and the omental bursa was opened. The left gastroepiploic vessels were cut. (e) The splenocolic ligament along the inferior spleen was then cut (red arrow). It was critical that the reverse side of the thin membrane of the transverse mesocolon was confirmed beforehand, via a bird's-eye view. (f) The confluence of the SMV, SPV, and PV was confirmed. (g) Branches from the SPV were detected before proceeding further (yellow arrows). A suitable dissection point for CHA skeletonization was also determined preoperatively (yellow circle). (h) Venous branches from the SPV were then skeletonized (yellow arrows). These venous branches were singly clipped and then cut by LCS. CHA, common hepatic artery; LCS: laparoscopic coagulation sheers; PV, portal vein; SMV, superior mesenteric vein; SPV, splenic vein.

A 75-year-old woman underwent computed tomography (CT) for follow-up assessment of the lung, and a pancreatic tumor was incidentally discovered. CT revealed a 4.0 cm tumor in the pancreatic tail. The tumor surface was severely calcified (Figure 1(a)), and therefore we did not attempt endoscopic ultrasound-guided fine needle aspiration (EUS-FNA). Positron emission tomography/CT showed no accumulation of fluorodeoxyglucose in the primary tumor. Endocrine and tumor marker levels were all within the normal ranges. Image studies including gadolinium-ethoxybenzyl-diethylenetriamine penta-acetic acid-enhanced magnetic resonance imaging (MRI) revealed no lymphoid or distant metastases. The tumor and anatomy were assessed in detail using a high-speed three-dimensional (3D) image analyzing system (Synapse Vincent, Fujifilm Medical, Tokyo, Japan). The tumor involved the splenic vein (SPV), but not the splenic artery (SPA) (Figures 1(b) and 1(c)). We also we ran simulations of our laparoscopic approach using this system and discussed the surgical procedures, preoperatively. Lymphadenectomy only for peripancreatic lymph nodes was proposed. Under general anesthesia, laparoscopic DP was begun with the patient in a supine position. Five ports were placed (Figure 1(d)). After establishing pneumoperitoneum at 10–12 mm Hg, a flexible electrolaparoscope was introduced through the intraumbilical port. The left gastroepiploic artery and vein were cut using an advanced bipolar device (ENSEAL G2 Tissue Sealers, Ethicon, Cincinnati, OH, USA), without clips. The inferior and superior ligaments surrounding the spleen were also cut (Figures 1(e)–1(h)). Dissection and removal of the distal pancreas were easily and effectively performed from a dorsal approach (Figures 2(a) and 2(b)), as in the preoperative simulation (Figure 2(c)). Although almost all procedures for mobilization of the pancreas can be completed using a dorsal approach, a ventral approach also allows safe removal of the pancreas (Figures 2(d)–2(f)). The pancreas was retracted using umbilical tape and/or was pushed aside using the shaft of the laparoscopic forceps (Figure 2(g)), followed by mobilization of the distal pancreas and spleen (Figures 2(g) and 2(h)). Based on the preoperative simulation for tumor location and anatomical landmarks (Figure 3(a)), intraoperative ultrasound (Arietta, Hitachi Aloka Medical, Tokyo, Japan) was performed to confirm the tumor location and to determine the transection line (Figure 3(b)). The SPA was ligated proximal to the cut line. Because we used a specific Covidien stapler (iDrive Ultra Powered Stapling System, Covidien, Dublin, Ireland), a preparatory surgical step for compression was required using an atraumatic clip to ensure safe stapling (Figures 3(c)–3(e)). For this stapling, we used a powered endostapler preloaded with an absorbable polyglycolic acid sheet (Endo GIA Reinforced Reload with Tri-Staple Technology, black cartridge, Covidien), based on our hypothesis that this cartridge may reinforce the pancreatic stump. The powered stapler was used to cut the pancreas and SPV* en bloc* (Figures 3(f)–3(h)). The specimen was then extracted through the 3 cm incision in a nonpermeable specimen pouch (Endo Catch Gold, Covidien) (Figure 4(a)). The capsule of the pancreas remnant was checked carefully, and a subtle injury to the capsule was seen near the staple line (Figure 4(b)). An abdominal drain was placed near the pancreas stump. The operative time was 3 hours and 31 minutes, and intraoperative blood loss was 30 mL. On gross section, the tumor was a solitary round mass with solid and cystic areas and hemorrhage and was bounded by a calcified capsule. Microscopically, surgical margins were adequate, and lymphoid metastasis was not detected. Although amylase levels in the drainage fluid increased slightly during the early postoperative period (Grade I in Clavien-Dindo classification and Grade A in the definition by International Study Group of Pancreatic Surgery), the postoperative course was essentially uneventful.

### 2.2. Case  2

**Figure 5:**
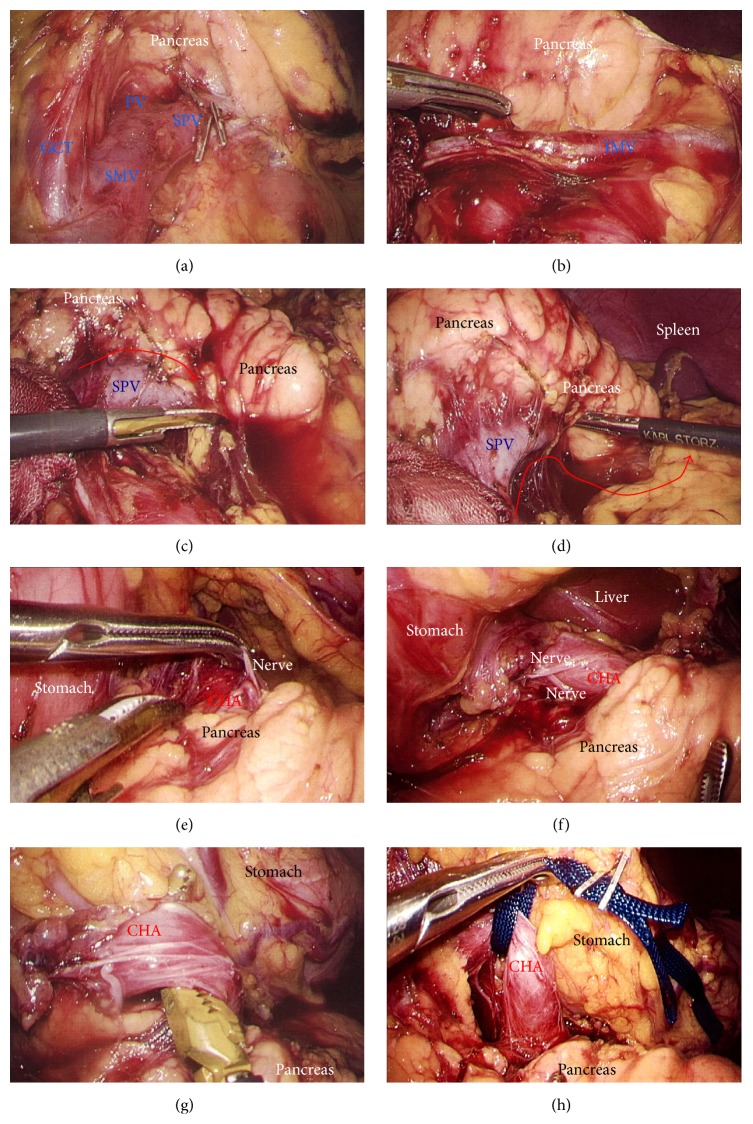
(a) The GCT was visualized. (b) The IMV was then skeletonized and preserved. (c) The SPV was skeletonized from the pancreatic parenchyma (red arrow). (d) The SPV was separated from dorsal fixation by connective tissue (red arrow). (e) and (f) The nerve surrounding the arterial sheath was useful to grasp the CHA without arterial injury. (g) and (h) The CHA was skeletonized and taped. CHA, common hepatic artery; GCT, gastrocolic trunk; IMV, inferior mesenteric vein; SPV, splenic vein.

**Figure 6:**
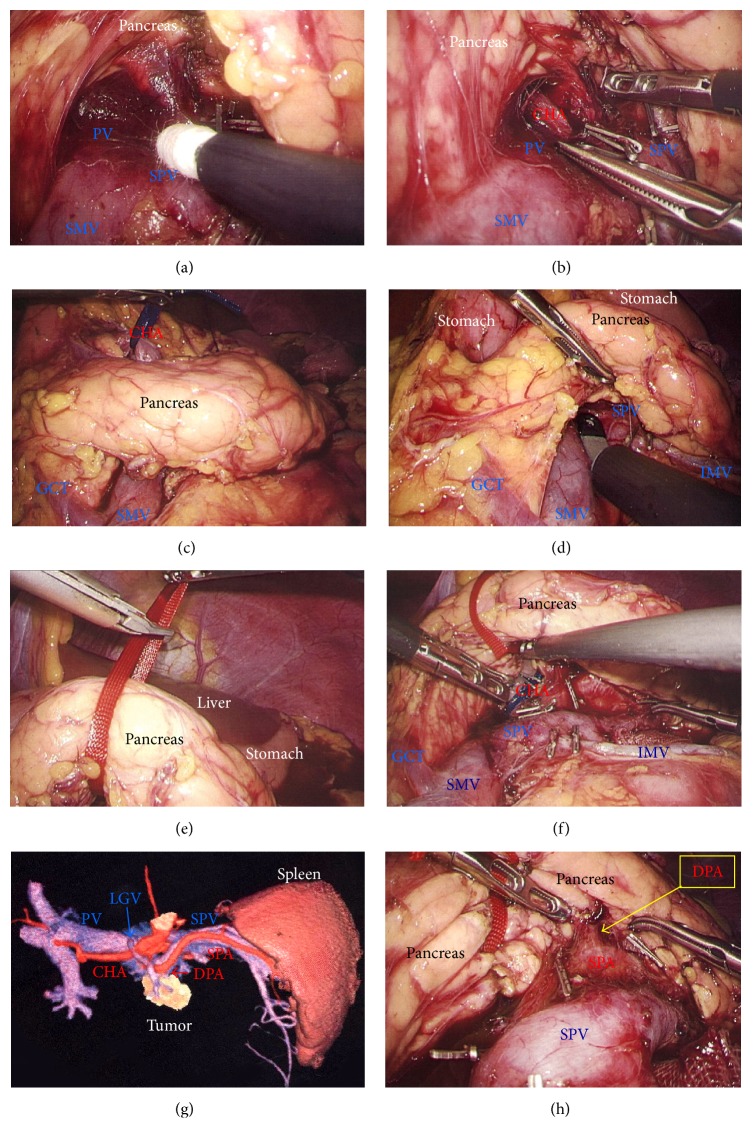
(a) The anterior wall of the SMV and PV was carefully and completely separated from the pancreatic parenchyma. (b) and (c) Tunneling of the pancreas was then performed at the level of the PV and SMV. (d) and (e) The pancreas was taped for retraction. (f) Sufficient tension was created using the forceps shaft. (g) Three-dimensional imaging revealed that the DPA branched from the SPA near the tumor. (h) The DPA was visualized. DPA, dorsal pancreatic artery; PV, portal vein; SMV, superior mesenteric vein; SPA, splenic artery.

**Figure 7:**
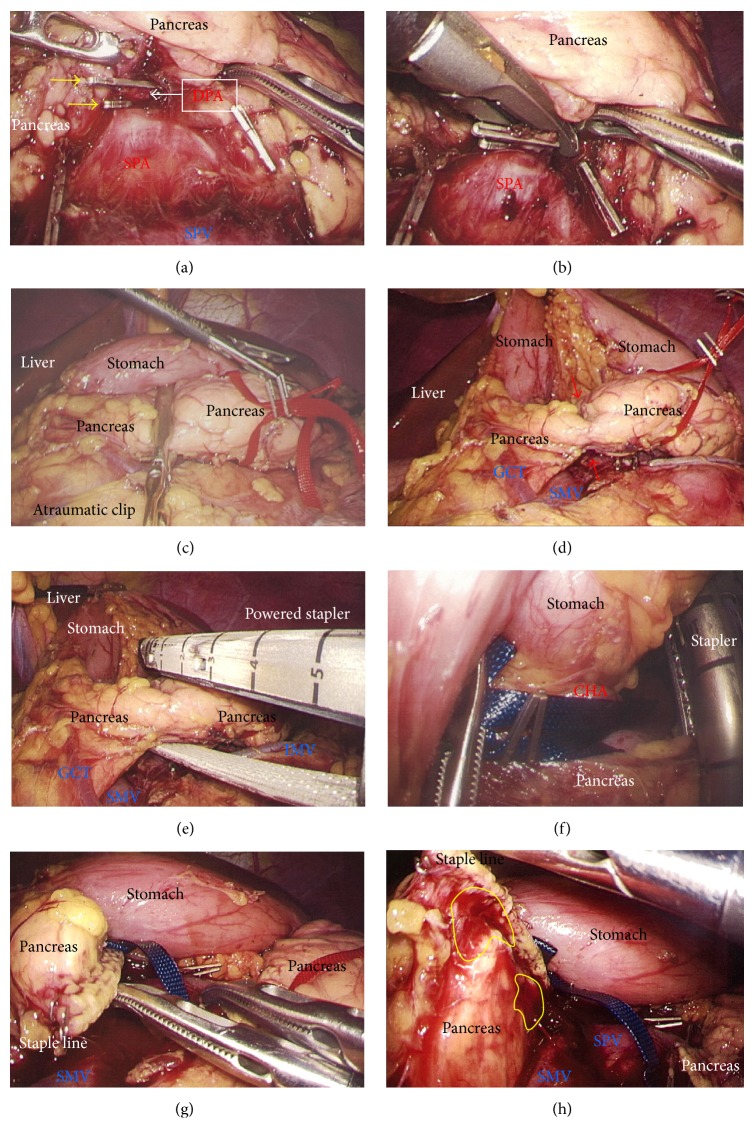
(a) The DPA was clipped twice with adequate margins from the SPA (yellow arrows). (b) The DPA was then cut using LCS. (c) Stapling was performed using the Covidien stapler with a separate and preceding compression step using an atraumatic clip. (d) Pancreatic parenchyma was compressed beforehand (red arrows). (e) A powered stapler was used to cut the pancreatic parenchyma. (f) Lack of involvement of vessels in the staple line was confirmed. (g) The pancreatic stump was examined. (h) The capsule of the pancreatic remnant was checked carefully and capsular injury was identified near the staple line (yellow circles). DPA, dorsal pancreatic artery; LCS, laparoscopic coagulation shears; SPA, splenic artery.

**Figure 8:**
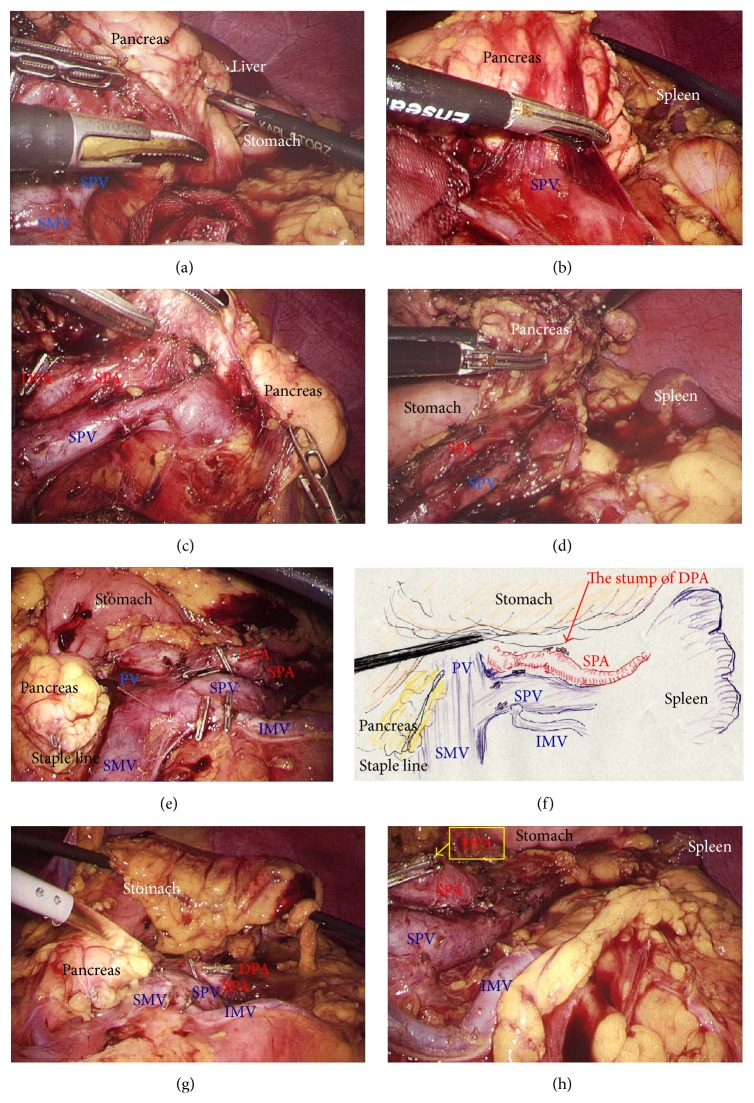
(a) and (b) The pancreatic body and tail were separated from the SPV. (c) and (d) The pancreatic body and tail were separated from the SPA. (e) and (f) The spleen was successfully preserved. (g) and (h) The local field was washed thoroughly and the pancreatic stump and vessel walls were checked carefully. SPA, splenic artery; SPV, splenic vein.

A 29-year-old woman underwent CT for gynecological examination, and a pancreatic tumor was discovered incidentally. CT revealed a 2.5 cm tumor in the body of the pancreas with both solid and cystic areas and calcification. Image studies (dynamic CT, MRI, and fluorodeoxyglucose-positron emission tomography/CT) revealed no enhancement in the primary tumor and no metastatic lesions. Lymphadenectomy only for peripancreatic lymph nodes was proposed. EUS-FNA was performed, and immunohistochemical testing for CD56, synaptophysin, *β*-catenin, progesterone receptor, CD10, galectin-3, chromogranin, and B-cell lymphoma/leukemia 10 was consistent with SPN. Tumor location and invasive findings were carefully assessed using the EUS finding and 3D image analysis (Figure 4(c)). We considered that both the SPA and SPV were separate from this tumor, and therefore preservation of the spleen was proposed. Gauze was placed over the splenic hilus before opening the omental bursa (Figure 4(d)). The reverse side of the thin membrane of the transverse mesocolon was confirmed via a bird's-eye view, and the inferior splenocolic ligament was then cut (Figure 4(e)). The anterior wall of the joint portion of the superior mesenteric vein (SMV), SPV, and portal vein (PV) was identified (Figure 4(f)). The 3D image revealed branches to the SPV (Figure 4(g)), which were then carefully skeletonized. These venous branches were singly clipped and then cut using ultrasonic laparoscopic coagulation shears (LCS) (Figure 4(h)). The gastrocolic trunk (GCT) (Figure 5(a)) and the inferior mesenteric vein (IMV) were identified next. Countertraction was applied to the pancreas avoiding venous injuries (Figure 5(a)) and the SPV was skeletonized both from the pancreatic parenchyma (Figure 5(c)) and from the dorsal fixation by connective tissue (Figure 5(d)). The location of the common hepatic artery (CHA) was assessed by 3D image (Figure 4(g)) and then skeletonized at its preoperatively planned point while avoiding injury to the vessels and the pancreas. The nerve near the arterial sheath was useful for grasping the CHA without causing arterial injuries (Figures 5(e) and 5(f)). The CHA was skeletonized (Figure 5(g)) and then taped (Figure 5(h)). Venous branches from the pancreas to the SMV and PV were assessed next. The anterior walls of the SMV and PV were carefully and completely detached from the pancreatic parenchyma (Figure 6(a)), and tunneling of the pancreas was done at the level of the PV and SMV (Figures 6(b) and 6(c)) freeing the pancreas to be taped (Figures 6(d) and 6(e)). For subsequent surgical procedures, the pancreas was retracted using the tape and/or was pushed aside using the shaft of the laparoscopic forceps (Figure 6(f)). The 3D image then revealed the dorsal pancreatic artery (DPA) branching from the SPA near the tumor (Figure 6(g)), detected at its preoperatively expected point (Figure 6(h)). This arterial branch was clipped twice (Figure 7(a)) and then cut by LCS (Figure 7(b)). Stapling was as in Case  1 and required a prestapling compression step using the atraumatic clip (Figures 7(c) and 7(d)). A powered stapler (iDrive Ultra Powered Stapling System, Endo GIA with Tri-Staple technology, black cartridge, Covidien) was then used to cut the pancreatic parenchyma (Figure 7(e)), avoiding injury to the vessels (Figure 7(f)). The specimen was extracted through the 3 cm incision in a specimen pouch (Endo Catch II, Covidien). The staple line of the pancreatic stump (Figure 7(g)) and the membrane of the pancreatic remnant (Figure 7(h)) were carefully examined. The pancreatic body and tail were then removed from the SPV (Figures 8(a) and 8(b)) and the SPA (Figures 8(c) and 8(d)), without any attached remnants of pancreatic parenchyma. Thus, the spleen was successfully preserved (Figures 8(e) and 8(f)). The local field was washed with warm saline (Figure 8(g)), and the pancreatic stump and vessel walls were then carefully examined (Figures 8(e) and 8(h)). An abdominal drain was placed near the pancreatic stump. The operative time was 5 hours and 20 minutes, and intraoperative blood loss was 20 mL. Histopathological findings revealed that the tumor had a heterogeneous appearance with solid cellular areas, pseudopapillary structures, hemorrhagic lesions, and necrotic debris. Cholesterol and hyaline globule deposits were observed, and no lymphoid metastasis was detected. Immunohistochemical findings were consistent with SPN and adequate surgical margins were reported. Severe leakage of pancreatic fluid was observed early in the postoperative period, and intravenous medications including antibiotics were required as intentional treatments for pancreatic leakage (Grade II in Clavien-Dindo classification and Grade B in the definition by International Study Group of Pancreatic Surgery). The patient was discharged on postoperative day 13.

## 3. Discussion

Pancreatic SPN is a rare neoplasm representing < 3% of pancreatic cancers [9–11]. The name of this entity dates to 1959 when Virginia Frantz first described a “papillary-cystic tumor of the pancreas” in the Armed Forces Institute of Pathology band on tumors of the pancreas [12]. The patient was a 2-year-old boy who died during an attempted pancreaticoduodenectomy. In 1970, Hamoudi et al. described the ultrastructural features of the tumor, which led to its acceptance as a separate clinicopathological entity [13]. Until its inclusion in the World Health Organization classification of pancreatic tumors in 1996 as “solid pseudopapillary tumor” of the pancreas, this condition has been described by different names in the literature including “papillary epithelial neoplasm of the pancreas,” “solid and cystic tumor of the pancreas,” “adenocarcinoma of the pancreas of childhood,” “papillary-cystic tumor,” and “solid and papillary epithelial neoplasm” [9]. In the current World Health Organization classification [14], SPN is defined as a low-grade malignant neoplasm of the exocrine pancreas. The term SPN has since gained wide acceptance and is currently the most frequently used name for this condition [15]. This likely reflects the increasing awareness of the clinical, pathological, and radiographic features of SPN and the uniformity of the nomenclature used for SPN in recent years. However, the etiology and differentiation status of SPN remain challenging and enigmatic [15]. Differential diagnoses for rare pancreatic tumors are important, and the immunocytochemical labeling pattern is very informative when diagnosing SPN, neuroendocrine tumors, acinar cell carcinomas, and pancreatoblastomas [16–19]. Usually, *β*-catenin [16, 17], *α*1-antitrypsin [16, 18, 19], progesterone receptor [16, 18, 19], CD10 [16, 18], CD99 [16], cytokeratin [16, 19], synaptophysin [16, 18, 19], and chromogranin [16, 19] are most important for immunocytochemistry.

SPN is classified as a low-grade exocrine pancreatic malignancy [14–16]. Metastasis occurs in 5–15% of cases [16] and lymph node metastasis occurs in 2% of cases [18]. Radiographic features have been summarized [16, 19] and typical findings on CT are a heterogeneous mass with solid and cystic areas, peripheral arterial enhancement of the solid area, and central calcification [20]. On MRI, SPN displays a heterogeneous signal in T1- and T2-weighted images, with low intensity in noncontrast T1-weighted images and high intensity in T2-weighted images [19]. EUS-FNA is important for preoperative diagnosis of SPN [16, 21], although the sensitivity and specificity of FNA are still not well defined [20]. EUS shows a well-demarcated cystic lesion with a solid component and calcification [16]. The first choice of treatments for SPN is surgery [16, 18, 22–24] and the role of chemotherapy and chemoradiation has not yet been established [16]. Postoperative recurrences occur in 4% of SPN patients at a mean time of 51 months [18].

Hepatobiliary pancreatic surgery represents the most challenging area in the field of digestive surgery, and pancreatic surgery has been historically associated with up to 50% morbidity and 5% mortality [25, 26]. Laparoscopic surgery is a revolutionary change in treatment [27–30]. In the field of pancreatic surgery, a porcine model of the initial surgical concepts was described in 1994 [31], and the first case series using these open surgical procedures was published in 1996 [32]. Laparoscopic pancreatic surgery was developed later [1–5, 33–39] and laparoscopic DP is now widely accepted around the world because it does not require anastomosis and other reconstructions [40, 41]. Many surgeons now consider laparoscopic DP a feasible and safe procedure [34, 35, 37, 38, 42] that is associated with less postoperative pain, faster recovery, and fewer wound-related complications. Overall, fewer general morbidity problems have also been documented, thus benefitting patients [34, 35, 43–47]; however, few surgeons have reported laparoscopic DP for SPN [6–8].

Intractable bleeding from the spleen by unexpected capsular injury is a concern. The reverse side of the thin membrane of the transverse mesocolon should be confirmed via a bird's-eye view before cutting the inferior splenocolic ligament. The mesocolon, adrenal glands, and Gerota's fascia are all retracted, creating the ideal surgical field for a dorsal approach with adequate mobilization of the spleen. In almost all cases, distal pancreas removal with skeletonization of the SPA can be performed by simply removing connective tissues via a dorsal approach (Figures 2(a) and 2(b)). A ventral approach can also permit complete and safe removal of the pancreas. To maintain the ideal surgical field, the surgical team should be well coordinated, and forceps must not cross during the procedures.

Laparoscopic DP is chosen mainly for patients with benign or low-grade malignancies, and patients with SPN are good candidates for laparoscopic DP [6, 7]. Some surgeons have documented laparoscopic DP with or without splenic preservation, for SPN [6–8]. Preserving the spleen or performing splenectomy is a topic of intense debate among proponents of the minimally invasive approach [48–50]. The rate of splenic conservation with laparoscopic DP is reported as 32–84% [45, 47, 51]. The successful rate of splenic preservation is higher with the laparoscopic approach [36, 45, 47]; however, splenic preservation should be carefully considered because even subtle residual tumor tissue left from preserving splenic vessels eliminates any oncological benefit [50]. The pancreatic parenchyma should be completely removed (Figure 8(b)) and the splenic vessels should be maintained without parenchymal remnants (Figures 8(c) and 8(d)). The better vision afforded by clear magnification with laparoscopy provides a higher rate of successful preservation of the spleen [34]. In our institution, we use a full high-definition system (Visera Elite, Olympus, Tokyo, Japan) and a flexible laparoscope with high light intensity (10 mm ENDOEYE, Olympus), to obtain clear magnification and a high contrast for successful laparoscopy.

The operative approach for splenic preservation should reflect the technical considerations including tumor location in the pancreas [50]. Splenic preservation with DP can be undertaken either with preservation or with sectioning of the splenic vessels, because blood flow to the spleen is maintained via the short gastric vessels. This procedure is called Warshaw's technique [52]; however, splenic infarction after laparoscopic DP with Warshaw's technique has been documented [53, 54]. A technical difficulty during the preservation of splenic vessels is the division of numerous short tributaries from the splenic vein spreading towards the pancreas [55]; this procedure requires special caution [55]. Appropriate use of modern technologies, such as electrothermal bipolar vessel sealers and LCS, achieves secure hemostasis of tributaries from the splenic vessels [34]. Additionally, although well-developed monopolar or bipolar devices are currently available, we believe that a novel fully integrated device with both ultrasonically generated frictional heat energy and electrically generated bipolar energy (Thunderbeat, Olympus) is an alternative for safe cutting, coagulation, and tissue dissection. To prepare for unexpected bleeding, devices for secure hemostasis and vessel sealing, such as a soft-coagulation system (VIO, Erbe, Tübingen, Germany), button-type electric pole with suction, self-irrigating monopolar (IO advanced, Erbe) coagulation, and bipolar thermofusion (BiClamp, Erbe), should be prepared preoperatively. Manipulation, such as rubbing a bleeding vessel using a button-type pole with suction and a soft-coagulation system, may also be effective for secure hemostasis.

Hand-assisted laparoscopic surgery has been used in laparoscopic DP [56, 57], according to surgeons' preferences [34, 58]. In our institution, we perform pure laparoscopic DP because hand-assisted laparoscopic surgery eliminates almost all of the advantages of laparoscopic surgery. Single incision laparoscopic DP has also been reported [59]. However, we do not employ a single incision approach because of increased risks and unfavorable limitations [60]. We perform laparoscopic DP in the supine position and therefore approach from the right side after cutting the pancreas. Although a left-sided approach may be advantageous for automatic shifting of the distal pancreas and spleen by gravity in the right lateral recumbent position, an approach from the right side after cutting the pancreas provides a well-coordinated use of forceps and scope in the supine position.

Pancreatic fistula formation after surgery remains the primary complication following pancreatic surgery [1, 61–65]. Management of the pancreatic stump is important [1, 61–70] and numerous important studies have been published to aid pancreatic surgeons in this task [1, 63–67, 69–75]. There is no difference between stapled and hand-sewn stamps [64, 69–71]; we use a stapler in laparoscopic DP. However, the question remains whether an orderly staple line ensures safer closure of the pancreatic stump. We have a clear impression that the neatness of the staple line is not associated with a safer stump; staplers close the pancreatic stamp in a fish-mouth shape. Pancreatic parenchyma and capsule should be adequately included in the staple line. Studies are ongoing to assess the hypothesis that a stapled stump is safer than a hand-closed stump. We originally suggested that studies should be designed as comparisons not between materials (i.e., stapler versus hand closure), but in technical safety (pancreatic parenchyma and membrane with or without injury). Even subtle excess tension during countertraction of the pancreas may result in unexpected capsular injuries. For stability during staple firing, we use a powered stapling device to attenuate excess countertraction as much as possible (http://www.ethicon.com/healthcare-professionals/products/staplers/endocutters/powered-echelon-flex#!overview). Staplers with a rounded floor as provided by Covidien require a separate compression step and may increase the risk of unexpected injury to the pancreatic parenchyma and/or capsule. In contrast, Ethicon staplers have a flat floor, permitting an all-inclusive one-step procedure. The Echelon Flex, Powered Endopath Stapler (Ethicon) provides safe and secure compression with stability in one step; fewer procedures decrease the risk of unexpected injuries.

**Figure 9:**
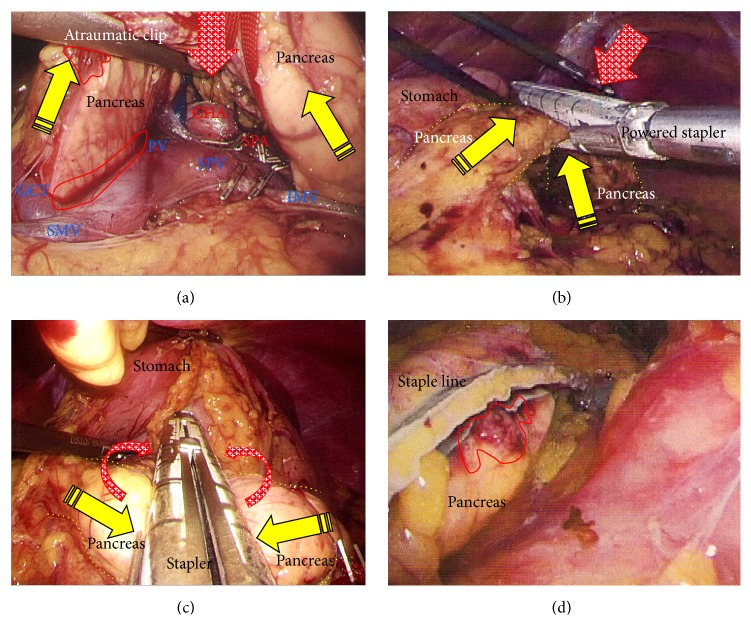
(a) Excess countertraction by the forceps and tape (yellow arrows) resulted in unexpected injury to the pancreatic parenchyma and capsule (red circles). Excess traction on the tape was released (red arrow). (b) Excess countertraction during stapling (yellow arrows) caused capsular injury near the staple line. Stapling should be performed as much to the dorsal side as possible to reduce excess countertraction (red arrow). A powered stapling system attenuates excess countertraction. (c) Excess tension when withdrawing during the stapler bite (yellow arrows) causes injury to the pancreatic parenchyma and membrane. All stapling procedures should be done at the correct position on the pancreas (red arrows). (d) Subtle injury to the capsule (red circle) should be never missed when evaluating the safety of pancreas resection.

Excess countertraction by the shaft of the forceps and/or excess tension when retracting the tape easily injures the pancreatic parenchyma and capsule. Staplers should hold the pancreas as steadily as possible, with minimal countertraction on the pancreas (Figures 9(a)–9(c)) and the pancreatic stump should be formed by adequate involution of the pancreatic capsule without causing injury. Optimal involution of the parenchyma and capsule is crucial for successful pancreatic stump formation. The pancreatic capsule should be carefully checked after being washed (Figures 4(b) and 8(g)); even subtle injury to the pancreatic parenchyma and capsule results in leakage of pancreatic fluid and subsequent intraperitoneal abscess (Figures 9(a) and 9(d)). For safe stapling, the stapler should be applied perpendicular to the pancreas. We use a 15 mm port for stapler insertion after direct confirmation of the pancreas, although this requires four ports to open the omental bursa (Figure 1(d)).

Although there are no definitive studies on the use of drains after laparoscopic DP, we usually place a closed drain at the pancreatic stump to monitor pancreatic secretion postoperatively. In our institution, this drain is usually removed within four days after surgery, and dynamic CT is routinely performed on postoperative day 7. Intraperitoneal puncture or endoscopic drainage is performed, if needed, based on the patient's clinical course, laboratory data, and image findings. Amylase and lipase levels in the drain discharge were very informative in our two cases. Drains should be placed automatically except in special circumstances such as with anticipated technical difficulties related to postoperative management of comorbid disorders.

In our institution, venous branches were singly clipped (Figure 4(h)) and arteries were dual-clipped (Figure 7(a)). These vessels were then cut using energy devices (Figure 7(b)). Note that arterial branches should be clipped at an adequate margin from the main arterial wall because handling near the main arterial wall may result in unexpected injury to the endothelium of the main artery after releasing the stapler. Because intensive dissections of the lymph nodes and arterial sheath are not required for SPN, the nerves around the arterial sheath are useful to grasp the arterial sheath without causing injury (Figures 5(a) and 5(b)). Specialized forceps, such as the “Mancina” forceps (a special-order forceps made by Olympus), are useful for grasping the nerve around the arterial sheath.

We contend that experience alone is insufficient for achieving safety in laparoscopic surgeries [27, 28]. Preoperative understanding of the planned procedure based on 3D image analysis is critical for successful laparoscopic surgery. In our institution, we routinely assessed the tumors and surgical anatomy for laparoscopic DP in each case, using a 3D image analyzer (Synapse Vincent, Fujifilm Medical). This system can detect the tumor location and depict surrounding tissues quickly, accurately, and safely. It also enables efficient planning of the operation settings if surgeons themselves create the simulation images. However, this simulation and preoperative discussion are still based on imagined scenarios by experienced surgeons, and the further development of a navigational system using real-time progress is needed.

Current laparoscopic instruments are well developed but each instrument should be used in the correct place and manner. There are a variety of available stapling devices and surgeons should follow the manufacturers' instructions to avoid possible malfunctions. Surgeons also must remain updated in their knowledge of how to use these devices. Many researchers have written systematic reviews of laparoscopic DP, and these reviews and institutional series described outcome parameters [76–80]. Many surgeons suggested that laparoscopic DP is safe and reasonable [76–80], though only few reports of laparoscopic DP for SPN were reported [6–8]. Laparoscopic DP with or without splenic preservation is beneficial for curative treatment of SPN if surgical procedures are carefully considered. Preoperative understanding and planning based on 3D image analysis are a powerful tool for successful laparoscopic DP, and the simulation and preoperative discussion are critical for oncological effectiveness and surgical safety.
